# BigDataScript: a scripting language for data pipelines

**DOI:** 10.1093/bioinformatics/btu595

**Published:** 2014-09-03

**Authors:** Pablo Cingolani, Rob Sladek, Mathieu Blanchette

**Affiliations:** ^1^McGill University School of Computer Science, 3480 University Street, Montreal, Québec H3A 0E9 and ^2^McGill University and Génome Québec Innovation Centre, 740 Dr. Penfield Avenue, Montréal, Québec H3A 0G1, Canada

## Abstract

**Motivation:** The analysis of large biological datasets often requires complex processing pipelines that run for a long time on large computational infrastructures. We designed and implemented a simple script-like programming language with a clean and minimalist syntax to develop and manage pipeline execution and provide robustness to various types of software and hardware failures as well as portability.

**Results:** We introduce the BigDataScript (BDS) programming language for data processing pipelines, which improves abstraction from hardware resources and assists with robustness. Hardware abstraction allows BDS pipelines to run without modification on a wide range of computer architectures, from a small laptop to multi-core servers, server farms, clusters and clouds. BDS achieves robustness by incorporating the concepts of absolute serialization and lazy processing, thus allowing pipelines to recover from errors. By abstracting pipeline concepts at programming language level, BDS simplifies implementation, execution and management of complex bioinformatics pipelines, resulting in reduced development and debugging cycles as well as cleaner code.

**Availability and implementation:** BigDataScript is available under open-source license at http://pcingola.github.io/BigDataScript.

**Contact:** pablo.e.cingolani@gmail.com

## 1 INTRODUCTION

Processing large amounts of data is becoming increasingly important and common in research environments as a consequence of technology improvements and reduced costs of high-throughput experiments. This is particularly the case for genomics research programs, where massive parallelization of microarray- and sequencing-based assays can support complex genome-wide experiments involving tens or hundreds of thousands of patient samples (Zuk *et al.*, 2014). With the democratization of high-throughput approaches and simplified access to processing resources (e.g. cloud computing), researchers must now routinely analyze large datasets. This paradigm shift with respect to the access and manipulation of information creates new challenges by requiring highly specialized skill, such as implementing data-processing pipelines, to be accessible to a much wider audience.

A data-processing pipeline, referred as ‘pipeline’ for short, is a set of partially ordered computing tasks coordinated to process large amounts of data. Each of these tasks is designed to solve specific parts of a larger problem, and their coordinated outcomes are required to solve the problem as a whole. Many of the software tools used in pipelines that solve big data genomics problems are CPU, memory or I/O intensive and commonly run for several hours or even days. Creating and executing such pipelines require running and coordinating several of these tools to ensure proper data flow and error control from one analysis step to the next. For instance, a processing pipeline for a sequencing-based genome-wide association study may involve the following steps (Auwera *et al.*, 2013): (i) mapping DNA sequence reads obtained from thousands of patients to a reference genome; (ii) identifying genetic changes present in each patient genome (known as ‘calling’ variants); (iii) annotating these variants with respect to known gene transcripts or other genome landmarks; (iv) applying statistical analyses to identify genetic variants that are associated with differences in the patient phenotypes; and (v) quality control on each of the previous steps. Even though efficient tools exist to perform each of these steps, coordinating these processes in a scalable, robust and flexible pipeline is challenging because creating pipelines using general-purpose computer languages (e.g. Java, Python or Shell scripting) involves handling many low-level process synchronization and scheduling details. As a result, process coordination usually depends on specific features of the underlying system’s architecture, making pipelines difficult to migrate. For example, a processing pipeline designed for a ‘multi-core server’ cannot directly be used on a cluster because running tasks on a cluster requires queuing them using cluster-specific commands (e.g. qsub). Therefore, if using such a language, programmers and researchers must spend significant efforts to deal with architecture-specific details that are not germane to the problem of interest, and pipelines have to be reprogrammed or adapted to run on other computer architectures. This is aggravated by the fact that the requirements change often and the software tools are constantly evolving.

In the context of bioinformatics, there are several frameworks to help implement data-processing pipelines; although a full comparison is beyond the scope of this article, we mention a few that relate to our work: (i) Snakemake (Köster and Rahmann, 2012) written as a Python domain-specific language (DSL), which has a strong influence from ‘make’ command. Just as in ‘make’, the workflow is specified by rules, and dependencies are implied between one rule’s input files and another rule’s output files. (ii) Ruffus (Goodstadt, 2010), a Python library, uses a syntactic mechanism based on decorations. This approach tends to spread the pipeline structure throughout the code, making maintenance cumbersome (Sadedin *et al.*, 2012). (iii) Leaf (Napolitano *et al.*, 2013), which is also written as a Python library, expresses pipelines as graphs ‘drawn’ using ASCII characters. Although visually rich, the authors acknowledge that this representation is harder to maintain than the traditional code. (iv) Bpipe (Sadedin *et al.*, 2012) is implemented as a DSL on top of Groovy, a Java Virtual Machine (JVM)-based language. Bpipe facilitates reordering, removing or adding pipeline stages, and thus, it is easy for running many variations of a pipeline. (v) NextFlow (www.nextflow.io), another Groovy-based DSL, is based on data flow programming paradigm. This paradigm simplifies parallelism and lets the programmer focus on the coordination and synchronization of the processes by simply specifying their inputs and outputs.

Each of these systems creates either a framework or a DSL on a pre-existing general-purpose programming language. This has the obvious benefit of leveraging the language’s power, expressiveness and speed, but it also means that the programmer may have to learn the new general-purpose programming language, which can be taxing and take time to master. Some of these pipeline tools use new syntactic structures or concepts (e.g. NextFlow’s data-flow programming model or Leaf’s pipeline drawings) that can be powerful, but require programming outside the traditional imperative model, and thus might create a steep learning curve.

In this article, we introduce a new pipeline programming language called BigDataScript (BDS), which is a scripting language designed for working with big data pipelines in system architectures of different sizes and capabilities. In contrast to existing frameworks, which extend general-purpose languages through libraries or DSLs, our approach helps to solve the typical challenges in pipeline programming by creating a simple yet powerful and flexible programming language. BDS tackles common problems in pipeline programming by transparently managing infrastructure and resources without requiring explicit code from the programmer, although allowing the programmer to remain in tight control of resources. It can be used to create robust pipelines by introducing mechanisms of lazy processing and absolute serialization, a concept similar to continuations (Reynolds, 1993) that helps to recover from several types of failures, thus improving robustness. BDS runs on any Unix-like environment (we currently provide Linux and OS.X pre-compiled binaries) and can be ported to other operating systems where a Java runtime and a GO compiler are available.

Unlike other efforts, BDS consists of a dedicated grammar with its own parser and interpreter, rather than being implemented on top of an existing language. Our language is similar to commonly used syntax and avoids inventing new syntactic structures or concepts. This results in a quick-to-learn, clean and minimalistic language. Furthermore, creating our own interpreter gives better control of pipeline execution and allows us to create features unavailable in general-purpose language (most notably, absolute serialization). This comes at the expense of expressiveness and speed. BDS is not as powerful as Java or Python, and our simple interpreter cannot be compared with sophisticated just-in-time execution or JVM-optimized byte-code execution provided by other languages. Nonetheless, in our experience, most bioinformatics pipelines rely on simple programmatic constructs. Furthermore, in typical pipelines, the vast majority of the running time is spent executing external programs, making the executing time of the pipeline code itself a negligible factor. For these reasons, we argue that BDS offers a good trade-off between simplicity and expressiveness or speed.

## 2 METHODS

In our experience, using general-purpose programming languages to develop pipelines is notably slow owing to many architecture-specific details the programmer has to deal with. Using an architecture agnostic language means that the pipeline can be developed and debugged on a regular desktop or laptop using a small sample dataset and deployed to a cluster to process large datasets without any code changes. This significantly reduces the time and effort required for development cycles. As BDS is intended to solve or simplify the main challenges in implementing, testing and programming data processing pipelines without introducing a steep learning curve, our main design goals are (i) simple programming language; (ii) abstraction from system’s architecture; and (iii) robustness to hardware and software failure during computationally intensive data analysis tasks. In the next sections, we explore how these concepts are implemented in BDS.

### 2.1 Language overview

BDS is a scripting language whose syntax is similar to well-known imperative languages. BDS supports basic programming constructs (if/else, for, while, etc.) and modularity constructs such as functions and ‘include’ statements, which are complemented with architecture-independent mechanisms for basic pipeline runtime control (such as task, sys, wait and checkpoint). At runtime, the BDS back-end engine translates these high-level commands into the appropriate architecture-dependent instructions. At the moment, BDS does not support object-oriented programming, which is indeed supported by other pipeline tools based on libraries/DSL extending general-purpose programming languages. The complete language specification and documentation is available online at http://pcingola.github.io/BigDataScript.

Unlike most scripting languages, BDS is strongly typed, allowing detection of common type conversion errors at the initial parsing stage (pseudo-compilation) rather than at runtime (which can happen after several hours of execution). As the syntax of strict typing languages tends to be more verbose owing to longer variable declaration statements, we provide a type inference mechanism (operator ‘:=’) that improves code readability. For example (Listing 1), the variables ‘in’ and ‘out’ are automatically assigned the types the first time they are used (in this case, the type is assigned to be string).

**Listing 1.**
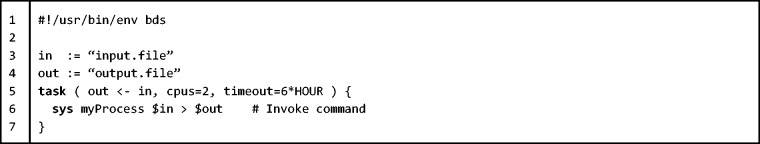
pipeline.bds program. A simple pipeline example featuring a task invoking a fictitious command ‘myProcess’ defined to require 2 CPUs and a maximum of 6 h of execution time (Line 5)

### 2.2 Abstraction from resources

One of the key features of BDS is that it provides abstraction from most architecture-specific details. In the same way that high-level programming languages such as C or Java allow abstraction of the CPU type and other hardware features, BDS supports system-level abstraction, including the number and the type of computing-nodes or CPU-cores that are available to the pipeline and its component tasks, whether firing another process may saturate the server’s memory or whether a process is executed immediately or queued.

Pipeline programming requires effective task management, particularly the ability to launch processes and wait for processes to finish execution before starting others. Task management can be performed using a single BDS statement, independently of whether this is running on a local computer or a cluster. Processes are executed using the task statement, which accepts an optional list of resources required by the task (for example, see Listing 1). The task consists of running a fictitious system command myProcess and diverting the output to ‘output.file’. BDS currently supports the following architectures: (i) local, single or multi-core computer; (ii) cluster, using GridEngine, Torque and Moab; (iii) server farm, using ssh access; and (iv) cloud, using EC2 and StarCluster. Depending on the type of architecture on which the script is run, the task will be executed by calling the appropriate queuing command (for a cluster) or by launching it directly (for a multi-core server).

BDS performs process monitoring or cluster queue monitoring to make sure all tasks end with a successful exit status and within required time limits. This is implemented using the ‘wait’ command, which acts as a barrier to ensure that no statement is executed until all tasks finished successfully. Listing 2 shows a two-step pipeline with task dependencies using a ‘wait’ statement (Line 13). If one or more of the ‘task’ executions fail, BDS will wait until all remaining tasks finish and stop script execution at the ‘wait’ statement. An implicit ‘wait’ statement is added at the end of the main execution thread, which means that a BDS script does not finish execution until all tasks have finished running. It is common for pipelines to need multiple levels of parallel execution; this can be achieved using the ‘parallel’ statement (or ‘par’ for short). Wait statements accept a list of task IDs/parallel IDs in the current execution thread.

**Listing 2.**
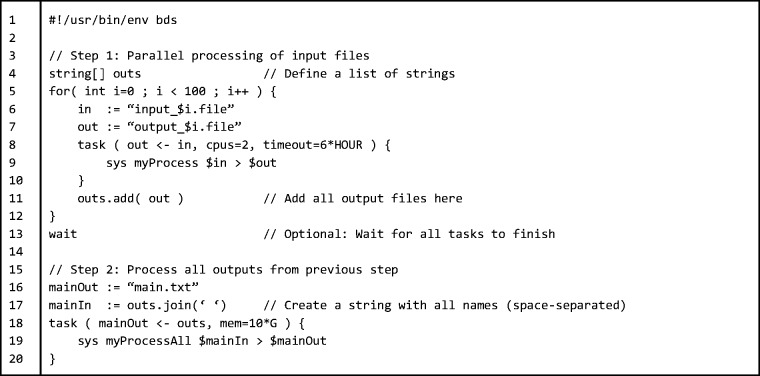
pipeline_2.bds program. A two-step pipeline with task dependencies. The first step (line 9) requires to run ‘myProcess’ command on a hundred input files, which can be executed in parallel. The second step (line 19) processes the output of those hundred files and creates a single output file (using fictitious ‘myProcessAll’ command). It should be noted that we never explicitly state which hardware we are using: (i) if the pipeline is run on a dual-core computer, as each process requires 2 CPUs, one ‘myProcess’ instance will be executed at the time until the 100 tasks are completed; (ii) if it is run on a 64-core server, then 32 ‘myProcess’ instances will be executed in parallel; (iii) if it is run on a cluster, then 100 ‘myProcess’ instances will be scheduled and the cluster resource management system will decide how to execute them; and (iv) if it is run on a single-core computer, execution will fail owing to lack of resources. Thus, the pipeline runs independent of the underlying architecture. The task defined in line 18 depends on all the outputs from tasks in line 8 (‘mainOut <− outs’)

In addition to supporting explicitly defined task dependencies, BDS also automatically models implicit dependencies using a directed acyclic graph (DAG) that is inferred from information provided in the dependency operators (‘<−’) contained in ‘task’ statements (see Listing 2, line 8). Finally, the ‘dep’ expression defines a task whose conditions are not evaluated immediately (as it happens in ‘task’ expressions) but only executed if required to satisfy a ‘goal’. Using ‘dep’ and ‘goal’ makes it easier to define pipelines in a ‘declarative’ manner that is similar to other pipeline tools, as tasks are executed only if the output needs to be updated with respect to the inputs, independent of the intermediate results file, which might have been deleted.

### 2.3 Robustness

BDS provides two different mechanisms that help create robust pipelines: lazy processing and absolute serialization. When a processing pipeline fails, BDS automatically cleans up all stale output files to ensure that rerunning the pipeline will produce a correct output. If a BDS program is interrupted, typically by pressing Ctrl-C on the console, all scheduled tasks and running jobs are terminated or deallocated from the cluster. In addition to immediately releasing computing resources, a clean stop means that users do not have to manually dequeue tasks, which allows them to focus on the problem at hand without having to worry about restoring a clean state.

**Lazy processing.** Complex processing pipelines are bound to fail owing to unexpected reasons that range from data format problems to hardware failures. Rerunning a pipeline from scratch means wasting days on recalculating results that have already been processed. One common approach, when using general-purpose scripting languages, is to edit the script and comment out some steps to save processing time, which is inelegant and error prone. A better approach is to develop pipelines that incorporate the concept of lazy processing (Napolitano *et al.*, 2013), a concept popularized by the ‘make’ command (Feldman, 1979) used to compile programs, and which simply means the work is not done twice. This concept is at the core of many of the pipeline programming tools, such as SnakeMake, Ruffus, Leaf and Bpipe. By design, when lazy processing pipelines are rerun using the same dataset, they avoid unnecessary work. In the extreme case, if a lazy processing pipeline is run on an already successfully processed dataset, it should not perform any processing at all.

BDS facilitates the creation of lazy processing pipelines by means of the dependency operator (‘<−’) and conditional task execution (see Listing 1, line 5 for an example). The task is defined as ‘task (out <− in)’, meaning that it is executed only if ‘out’ file needs to be updated with respect to ‘in’ file: for example, if ‘output.file’ file does not exist, has zero length, is an empty directory or has been modified before ‘input.file’.

**Absolute serialization.** This refers to the ability to save and recover a snapshot of the current execution state, compiled program, variables, scopes and program counter, a concept similar to ‘con-tinuations’ (Reynolds, 1993). BDS can perform an absolute serialization of the current running state and environment, producing ‘checkpoint files’ from which the program can be re-executed, either on the same computer or on any other computer, exactly from the point where execution terminated. Checkpoint files (or ‘checkpoints’ for short) also allow all variables and the execution stack to be inspected for debugging purposes (‘bds -i checkpoint.chp’). The most common use of checkpoints is when a task execution fails. On reaching a ‘wait’ statement, if one or more tasks have failed, BDS creates a checkpoint, reports the reasons for task execution failure and terminates. Using the checkpoint, pipeline execution can be resumed from the point where it terminated (in this case, at the most recently executed ‘wait’ statement) and can properly re-execute pending tasks (i.e. the tasks that previously failed execution).

**Limitations.** BDS is designed to afford robustness to the most common types of pipeline execution failures. However, events such as full cluster failures, emergency shutdowns, head node hardware failures or network problems isolating a subset of nodes may result in BDS being unable to exit cleanly, leading to an inconsistent pipeline state. These problems can be mitigated by a special purpose ‘checkpoint’ statement that, as the name suggests, allows the programmer to explicitly create checkpoints. Given that the overhead of creating checkpoints is minimal (a few milliseconds compared with hours of processing time for a typical pipeline), carefully crafted checkpoint statements within the pipeline code can be useful to prevent losing processed data, mitigate damage and minimize the overhead when rerun, which can be critical for long running pipelines.

### 2.4 Other features

Here we mention some selected features that are useful in pipeline programming. Extensive documentation is available at http://pcingola.github.io/BigDataScript*.*

**Fig. 1.**
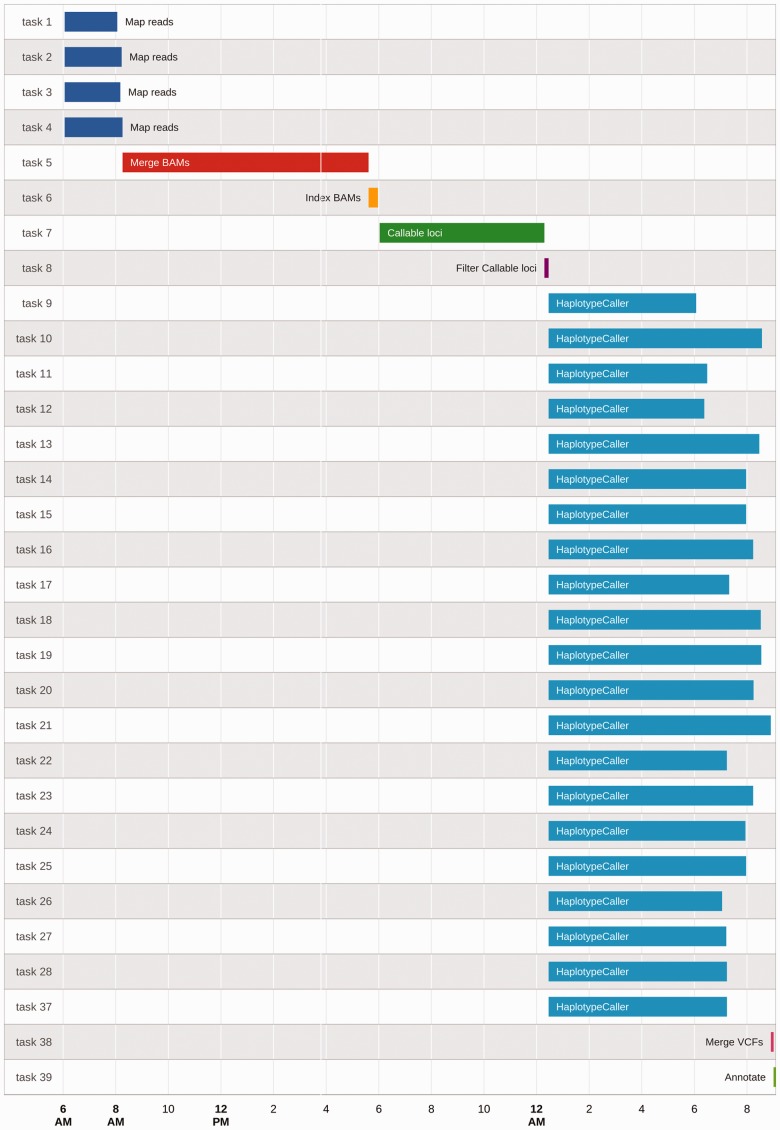
BDS report showing pipeline’s task execution timeline

**Automatic logging.** Logging all actions performed in pipelines is important for three reasons: (i) it helps debugging; (ii) it improves repeatability; and (iii) it performs audits in cases where detailed documentation and logging are required by regulatory authorities (such as clinical trials). Creating log files is simple, but it adds boilerplate code and increases the complexity of the pipeline. BDS performs automatic logging in three different ways. First, it directs all process StdOut/StdErr output to the console. Second, as having a single output can be confusing when dealing with thousands of processes running in parallel, BDS automatically logs each process’s outputs (StdOut and StdErr) and exit codes in separate clearly identified files. Third, BDS creates a report showing both an overview and details of pipeline execution (Fig. 1).

**Automatic command line parsing.** Programming flexible data pipelines often involves parsing command-line inputs—a relatively simple but tedious task. BDS simplifies this task by automatically assigning values to variables specified through the command line. As an example, if the program in Listing 1 is called ‘pipeline.bds’, then invoking the program as ‘pipeline.bds -in another.file’ will automatically replace the value of variable ‘in’ with ‘another.file’.

**Task re-execution.** Tasks can be re-executed automatically on failure. The number of retries can be configured globally (as a command-line argument) or by a task (using the ‘retry’ variable). Only after failing ‘retry+1’ times will a task will be considered to have failed.

### 2.5 BDS implementation

BDS is programmed using Java and GO programming languages. Java is used for high-level actions, such as performing lexical analysis, parsing, creating abstract syntax trees (AST), controlling AST execution, serializing processes, queuing tasks, etc. Low-level details, such as process execution control, are programmed in GO. As BDS is intended to be used by programmers, it does not rely on graphical interfaces and does not require installation of complex dependencies or Web servers.

**Fig. 2.**
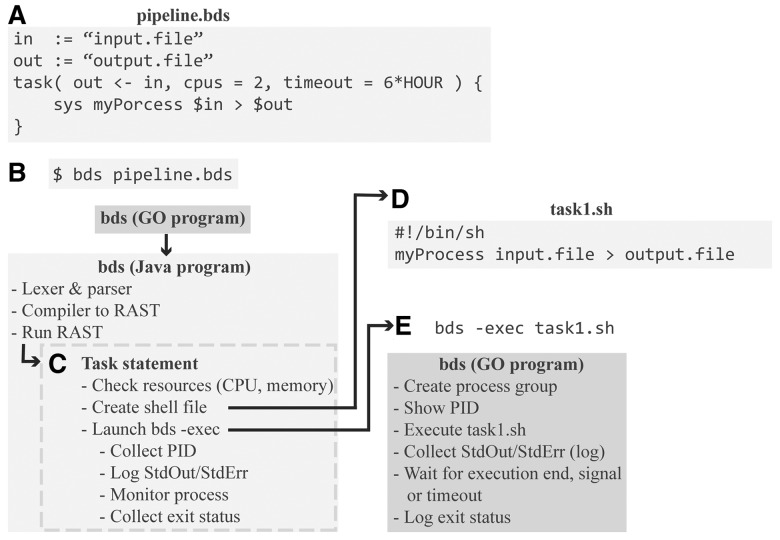
Execution example. (**A**) Script ‘pipeline.bds’. (**B**) The script is executed from a terminal. The GO executable invokes main BDS, written in JAVA, performs lexing, parsing, compilation to AST and runs AST. (**C**) When the task statement is run, appropriate checks are performed. (**D**) A shell script ‘task1.sh’ is created, and a bds-exec process is fired. (**E**) bds-exec reports PID, executed the script ‘task1.sh’ while capturing stdout and stderr as well as monitoring timeouts and OS signals. When a process finishes execution, the exit status is logged

Figure 2 shows the cascade of events triggered when a BDS program is invoked. First the script pipeline.bds (Fig. 2A) is compiled to an AST structure (Fig. 2B) using ANTLR (Parr, 2007). After creating the AST, a runnable-AST (RAST) is created. RAST nodes are objects representing statements, expressions and blocks from our BDS implementation. These nodes can execute BDS code, serializing their state, and recover from a serialized file, thus achieving absolute serialization. The script is run by first creating a scope and then properly traversing the RAST (Fig. 2C). We note that if needed, this approach could be tuned to perform efficiently, as demonstrated by modern languages, such as Dart.

When recovering from a checkpoint, the scopes and RAST are de-serialized (i.e. reconstructed from the file) and then traversed in ‘recovery mode’, meaning that the nodes do not execute BDS code. When the node that was executed at the time of serialization event is reached, BDS switches to ‘run mode’ and the execution continues. This achieves execution recovery from the exact state at serialization time. Checkpoints are the full state of a program’s instance and are intended as a recovery mechanism from a failed execution. This includes failures owing to corrupted or missing files, as BDS will re-execute all failed tasks when recovering, thus correcting outputs from those tasks. However, checkpoints are not intended to recover from programming errors, where the user modifies the program to fix a bug, as a previously generated checkpoint is no longer valid respect to the new source code.

When a task statement is invoked, process requirements, such as memory, CPUs and timeouts, can optionally be specified. Depending on the architecture, BDS either checks that the underlying system has appropriate resources (CPUs and memory) to run the process (e.g. local computer or ssh-farm) or relies on the cluster management system to appropriately allocate the task. If all task requirements are met, a script file is created (Fig. 2D), and the task is executed by running an instance of bds-exec, a program that controls execution (Fig. 2E). This indirection is necessary for five reasons, which are described in detail below: (i) process identification, (ii) timeout enforcement, (iii) logging, (iv) exit status report and (v) signal handling.

**Process identification** means that bds-exec reports its process ID (PID), so that BDS can kill all child processes if the BDS script execution is terminated for some reason (e.g. the Ctrl-C key is pressed at the console).

**Timeout enforcement** has to be performed by bds-exec as many underlying systems do not have this capability (e.g. a process running on a server). When a timeout occurs, bds-exec sends a kill signal to all child processes and reports a timeout error exit status that propagates to the user terminal and log files.

**Logging** a process means that bds-exec redirects stdout and stderr to separate log files. These files are also monitored by the main BDS process, which shows the output on the console. As there might be thousands of processes running at the same time and operating systems have hard limits on the number of simultaneous file descriptors available for each user, opening all log files is not an option. To overcome this limit, BDS polls log file sizes, only opening and reading the ones that change.

**Exit status** has to be collected to make sure a process finished successfully. Unfortunately, there is no unified way to do this, and some cluster systems do not provide this information directly. By saving the exit status to a file, bds-exec achieves two goals: (i) unified exit status collection and (ii) exit status logging.

**Signal handling** is also enforced by bds-exec making sure that a kill signal correctly propagated to all subprocesses, but not to parent processes. This is necessary because there is no limit on the number of indirect processes that a task can run, and Unix/Posix systems do not provide a unified way to obtain all nested child processes. To be able to keep track of all subprocesses, bds-exec creates a process group and spawns the subprocess in it. When receiving a signal from the operating system, bds-exec traps the signal and propagates a kill signal to the process group.

## 3 RESULTS

To illustrate the use of BDS in a real-life scenario, we present an implementation of a sequencing data analysis pipeline. This example illustrates three key BDS properties: architecture independence, robustness and scalability. The data we analyzed in this example consist of high-quality short-read sequences (200× coverage) of a human genome corresponding to a person of European ancestry from Utah (NA12877), downloaded from Illumina platinum genomes (http://www.illumina.com/platinumgenomes).

**Fig. 3.**
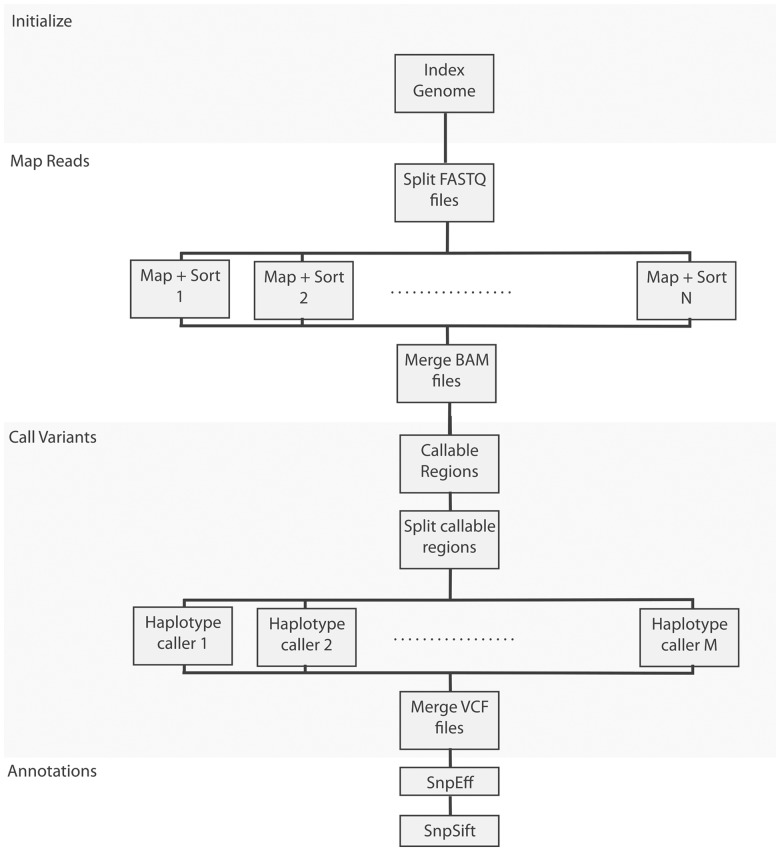
Whole-genome sequencing analysis pipeline’s flow chart, showing how computations are split across many nodes

The example pipeline we created follows current best practices in sequencing data analysis (McKenna *et al.*, 2010), which involves the following steps: (i) map reads to a reference genome using BWA (Li and Durbin, 2009), (ii) call variants using GATK’s HaplotypeCaller and (iii) annotate variants using SnpEff (Cingolani *et al.*, 2012b) and SnpSift (Cingolani *et al.*, 2012a). The pipeline makes efficient use of computational resources by making sure tasks are parallelized whenever possible. Figure 3 shows a flowchart of our implementation, while the pipeline’s source code is available at ‘include/bio/seq’ directory of our project’s source code (https://github.com/pcingola/BigDataScript).

**Architecture independence.** We ran the exact same BDS pipeline on (i) a laptop computer; (ii) a multi-core server (24 cores, 256 GB shared RAM); (iii) a server farm (5 servers, 2 cores each); (iv) a 1200-core cluster; and (v) the Amazon AWS Cloud computing infrastructure (Table 1). For the purpose of this example and to accommodate the fact that running the pipeline on a laptop using the entire dataset would be prohibitive, we limited our experiment to reads that map to chromosome 20. The architectures involved were based on different operating systems and spanned about three orders of magnitude in terms of the number of CPUs (from 4 to 1200) and RAM (from 8 GB to 12 TB). BDS can also create a cluster from a ‘server farm’ by coordinating raw SSH connections to a set of computers. This minimalistic setup only requires that the computers have access to a shared disk, typically using NFS, which is a common practice in companies and university networks.

**Table 1.** Architecture independence example

System	CPUs	RAM	Notes
Laptop (OS.X)	4	8 GB	
Server (Linux)	24	256 GB	
Server farm (ssh)	16	8 Gb	Server farm using 8 nodes, 2 cores each.
Cluster (PBS Torque)	1200	12 TB	High load cluster (over 95%).
Cluster (MOAB) (Random failures)	1200	12 TB	High load cluster (over 95%). Hardware induced failures.
Cloud (AWS + SGE)	Inf.	Inf.	StarCluster, 8 m1.large instances.

*Notes:* Running the same BDS-based pipeline, a sequence variant calling and analysis pipeline, on the same dataset (chr20) but different architectures, operating systems and cluster management systems.

In all cases, the overhead required to run the BDS script itself accounted for <2 ms per task, which is negligible compared with typical pipeline runtimes of several hours.

**Robustness.** To assess BDS’s robustness, we ran the pipeline on a cluster where ∼10% of the nodes have induced hardware failures. As opposed to software failures, which are usually detected by cluster management systems, hardware node failures are typically more difficult to detect and recover from. In addition, we elevated the cluster load to >95% to make sure the pipeline was running on less than ideal conditions. As shown in Table 1, the pipeline finished successfully without any human intervention and required only 30% more time than in the ideal case scenario because BDS had to rerun several failed tasks. This shows how BDS pipelines can be robust and recover from multiple failures by using lazy processing and absolute serialization mechanisms.

**Scalability.** To assess BDS’s scalability, we ran exactly the same pipeline on two datasets that vary in size by several orders of magnitude (Table 2): (i) a relatively small dataset (chromosome 20 subset, ∼2 GB) that would typically be used for development, testing and debugging and (ii) a high-depth whole-genome sequencing dataset (over 200× coverage, roughly 1.5 TB).

**Table 2.** Scaling dataset sized by a factor of ∼1000

Dataset	Dataset size	System	CPUs	RAM
chr20	2 GB	Laptop (OS.X)	4	8 GB
Whole genome	1.5 TB	Cluster (MOAB)	22 000	80 TB

*Notes:* The same sample pipeline run on dataset of 2 GB (reads mapping to human chromosome 20) and 1.5 TB (whole-genome data set). Computational times vary according to system’s resources, utilization factor and induced hardware failures.

## 4 DISCUSSION

We introduced BDS, a programming language that simplifies implementing, testing and debugging complex data analysis pipelines. BDS is intended to be used by programmers in a similar way to shell scripts, by providing ‘glue’ for several tools to ensure that they execute in a coordinated way. Shell scripting was popularized when most personal computers had a single CPU and clusters or clouds did not exist. One can thus see BDS as extending the hardware abstraction concept to data-center level while retaining the simplicity of shell scripting.

BDS tackles common problems in pipeline programming by abstracting task management details at the programming language level. Task management is handled by two statements (‘task’ and ‘wait’) that hide system architecture details, leading to cleaner and more compact code than general-purpose languages. BDS also provides two complementary robustness mechanisms: lazy processing and absolute serialization.

A key feature is that being architecture agnostic, BDS allows users to code, test and debug big data analysis pipelines on different systems than the ones intended for full-scale data processing. One can thus develop a pipeline on a laptop and then run exactly the same code on a large cluster. BDS also provides mechanisms that eliminate many boilerplate programming tasks, which in our experience significantly reduce pipeline development times. BDS can also reduce CPU usage, by allowing the generation of code with fewer errors and by allowing more efficient recovery from both software and hardware failures. These benefits generally far outweigh the minimal overhead incurred in typical pipelines.
